# CD8+ T Cell-Mediated Mechanisms Contribute to the Progression of Neurocognitive Impairment in Both Multiple Sclerosis and Alzheimer's Disease?

**DOI:** 10.3389/fimmu.2020.566225

**Published:** 2020-11-19

**Authors:** Zorica Stojić-Vukanić, Senka Hadžibegović, Olivier Nicole, Mirjana Nacka-Aleksić, Sanja Leštarević, Gordana Leposavić

**Affiliations:** ^1^Department of Microbiology and Immunology, University of Belgrade-Faculty of Pharmacy, Belgrade, Serbia; ^2^Institut des Maladies Neurodégénératives, CNRS, UMR5293, Bordeaux, France; ^3^Institut des Maladies Neurodégénératives, Université de Bordeaux, UMR5293, Bordeaux, France; ^4^Department of Pathobiology, University of Belgrade-Faculty of Pharmacy, Belgrade, Serbia

**Keywords:** multiple sclerosis, Alzheimer's disease, neurocognitive impairment, effector/memory CD8+ T cells, CD8+ tissue-resident memory T cells, microglia

## Abstract

Neurocognitive impairment (NCI) is one of the most relevant clinical manifestations of multiple sclerosis (MS). The profile of NCI and the structural and functional changes in the brain structures relevant for cognition in MS share some similarities to those in Alzheimer's disease (AD), the most common cause of neurocognitive disorders. Additionally, despite clear etiopathological differences between MS and AD, an accumulation of effector/memory CD8+ T cells and CD8+ tissue-resident memory T (Trm) cells in cognitively relevant brain structures of MS/AD patients, and higher frequency of effector/memory CD8+ T cells re-expressing CD45RA (TEMRA) with high capacity to secrete cytotoxic molecules and proinflammatory cytokines in their blood, were found. Thus, an active pathogenetic role of CD8+ T cells in the progression of MS and AD may be assumed. In this mini-review, findings supporting the putative role of CD8+ T cells in the pathogenesis of MS and AD are displayed, and putative mechanisms underlying their pathogenetic action are discussed. A special effort was made to identify the gaps in the current knowledge about the role of CD8+ T cells in the development of NCI to “catalyze” translational research leading to new feasible therapeutic interventions.

## Introduction

Neurocognitive impairment (NCI) is an important feature of multiple sclerosis (MS) and might be even more relevant to patients than mobility restrictions (1). More important, there is no efficient therapy for the NCI (2, 3). Hence, it is important to incorporate cognitive assessment into MS clinics and to stimulate research leading to effective interventions to moderate the NCI (4). To “catalyze” this research, we attempted to identify the major gaps in the current understanding of the immunopathogenesis of NCI. In this attempt, we considered that despite clear etiological differences, MS as Alzheimer's disease (AD) may be presented with dementia (major neurocognitive disorder in *DSM-5* classification) (5–7), and that some similar immunohistopathological changes are found in cognitively relevant brain structures of MS and AD patients (8–12). Besides innate immunity cell-mediated neuroinflammation (13–15), accumulating evidence indicates involvement of the adaptive cellular immunity in the pathogenesis of not only MS but also AD. Although CD4+ T cells are shown to be the major driver of MS pathogenesis (16–19) and to be implicated in AD development (19–21), therapeutic strategies selectively altering/inhibiting the function of CD4+ T cells showed disappointing results in MS and AD alike (20, 22, 23). On the other hand, there are data indicating that (i) CD8+ T cells are the predominant type of T cells in MS brain lesions (8–10) and (ii) NCI development in AD patients and transgenic mouse AD models coincides with CD8+ T cells' infiltration into cognitively relevant brain structures (11, 12, 24–27). Thus, it may be hypothesized that CD8+ T cells contribute to the development of NCI in MS and AD. In this mini-review, the results from a side-by-side comparative analysis of literature data corroborating the role of CD8+ T cells are displayed as a starting point for translational research leading to feasible therapeutic interventions.

## NCI And Histopathological Signature of Ms and Ad

NCI is seen in 43–70% of people diagnosed with MS (28); in the subclinical radiologically isolated syndrome, clinically isolated syndrome, and all phases of clinical MS (29). It is also detected in experimental autoimmune encephalomyelitis (EAE), the often used animal model of MS (30–36). The most commonly impaired cognitive domains in MS include memory, attention, executive functions, speed of information processing, and visual-spatial abilities (37). The neurocognitive profiles of MS patients substantially differ depending on (i) clinical subtype and duration of the disease (38–41); (ii) age, sex, and ethnicity (38–41); (iii) brain (reflects maximal lifetime brain volume determined by genetics) and cognitive (gains throughout life experience, e.g., education, intellectually enriching leisure activities) reserve (42); and (iv) differences in screening tools used for neurocognitive evaluation (41). A proportion of MS patients meets the criteria for dementia (5, 7). On the other hand, AD is shown to be the most common cause of dementia, accounting for 60–80% of dementia cases (www.alz.org/alzheimers-dementia). All cognitive domains may be affected in AD patients (43). The neurocognitive profile of AD patients depends on stage of the disease and brain and cognitive reserves (44). In the development of NCI in AD, changes corresponding to mild cognitive disorder in *DSM-5* classification may also be detected (45). Of note, when cognitive functions in older MS patients were evaluated, similarities between their neurocognitive profiles and those of patients with mild cognitive disorder of the AD type (anamnestic mild cognitive impairment) were found (40).

In MS patients apart from characteristic inflammatory-demyelinating lesions of the white matter, inflammation and neurodegeneration in cortical and deep gray matter, which are associated with deficits in learning and memory in AD, was found (9, 46, 47). On the other hand, although AD is characterized by the gray matter damage, disruption of white matter integrity was also described (48).

The development of typical brain lesions in AD is linked with the neurotoxic β-amyloid peptide (Aβ) variants that form soluble oligomers and insoluble fibers, and Aβ-induced hyperphosphorylation of the microtubule-associated protein tau (49–52). Thus, in hippocampal and cortical regions of AD patients, extracellular aggregates of amyloid fibrils—senile plaques and intracellular aggregates of hyperphosphorylated tau—neurofibrillary tangles are typically present (49–52). Although aggregation of oligomeric Aβ (oAβ) and plaques formation is a major feature of AD (53), soluble oAβ, particularly those encompassing Aβ_1−42_, are neurotoxic (54). On the other hand, in MS despite the augmented expression of the amyloid precursor protein (APP), reflecting axonal damage (55, 56), and the increased levels of soluble α-APP and β-APP, intermediate products of APP proteolysis, in brain lesions (57), amyloid plaques have not been found (58–62). The latter could reflect an enhanced demyelinization and release of myelin basic protein, as this protein inhibits amyloid fibril formation (favoring the detrimental effect of their soluble precursors) (63–66) and/or their enhanced cleaning due to microglial activation (62). However, as TNF-α, a major proinflammatory cytokine, impairs autophagic flux of Aβ aggregates in microglia (67), the latter does not seem likely. Additionally, it is supposed that the generation/clearance of (o)Aβ in MS varies during the disease progression or depending on the phase of the disease (68, 69). This may explain the discrepancies between data on their concentration in cerebrospinal fluid (CSF) (70–78). Of note, although alterations in Aβ metabolism are implicated in the impairment of neural plasticity and the development of NCI in MS (79, 80), there are no data on concentration of neurotoxic (o)Aβ in brain tissue. Differently from amyloid plaques, characteristic insoluble hyperphosphorylated tau formation has been described in the brain in the neurodegenerative phase of EAE and MS (81–83).

## Structural and Functional Alterations of Synapses

It appears that synaptic loss precedes neuronal loss in AD, and these effects are probably driven by amyloid and tau pathology (52, 84). Post-mortem analyses of synapses/synaptic markers (synapsin I, synaptophysin, and post-synaptic density protein 95) in hippocampal and frontotemporal tissue provide evidence for strong synapse loss in MS/EAE as well (32, 85–88). The synaptic loss is suggested to be the strongest correlate of NCI in AD (89–91). In EAE, a positive correlation between hippocampal-dependent memory impairment and synaptic loss was also found (32, 92).

While the density of synapses is a key determinant to control the complexity and diversity of neuronal networks, the ability of neurons to durably strengthen their connections, also called synaptic plasticity, is crucial to shape the neuronal networks necessary for learning and memory [reviewed in (93)]. Long-term potentiation (LTP) and long-term depression (LTD) are two forms of synaptic plasticity and leading candidates for mechanisms underlying learning and memory (94). Generally, in experimental models of AD soluble oAβ are shown to cause LTP impairment, as well as LTD depression and synaptic loss [reviewed in (95)]. Although LTP impairment is also found in EAE (96–101), there is no direct evidence for a role of soluble oAβ [reviewed in (95)].

It is likely that several post-synaptic receptors mediate soluble oAβ toxicity at the post-synaptic compartment (102). However, NMDA receptors (NMDARs) seem to be particularly important in both animal AD (103) and MS models (97). The alteration of synaptic plasticity in AD models has been linked with oAβ-induced increase in glutamate concentration at synapses (85, 104, 105) and its “spillover” out of the synaptic clefts (106). The latter causes recruitment of extrasynaptic NMDARs (107–109) and neuronal excitotoxicity (110, 111) resulting in progressive neuronal and memory loss (106). Additionally, specific activation of extrasynaptic NMDARs in animal AD models enhances the amyloidogenesis and Aβ release (112) and tau phosphorylation (106, 113–115), leading to a vicious circle and the disease progression (116). Thus, there is a positive correlation between oAβ release, tau pathology, and NCI in AD (117). On the other hand, the role of oAβ in the development of NCI in MS (79, 80) requires additional research.

### Research Challenges

To further investigate the role of oAβ/phosphorylated tau in the development of NCI in MS/EAE.

## CD8+ T Cells In Ms and Ad Lesions

### CD8+ T Cells in MS Lesions

In MS lesions, including those relevant for NCI, the vast majority of CD3+ T cells were found to be clonally expanded CD8+ T cells (10, 118–124). Their number correlates with the severity of axonal damage (125). In NCI-related brain lesions of EAE-affected animals, T cells were also detected (36, 126), and the demyelination was shown to be more MHC class I- than MHC class II-dependent (127), suggesting an active role for CD8+ T cells in the destructive CNS immune response. To harmonize these findings with the long-standing view of MS as a CD4+ T-cell-driven disease (128), it was hypothesized that following the disease initiation CD4+ T cells (the key drivers of the disease initiation) in MS/EAE are eliminated by apoptosis (124, 129), so CD8+ T cells take on a leading role (130, 131). Furthermore, the genetics (HLA A^*^0301 and HLA A^*^0201 was associated with a higher risk for MS and a protective effect on MS, respectively) (132, 133) evinces CD8+ T cell involvement in MS (134). Consistently, in active MS lesions (125, 135) and CSF (136–138) classical cytotoxic CD8+ T lymphocytes (CTLs) were found. Of note, CTLs with polarized perforin/granzyme granules were observed in close proximity to oligodendrocytes/demyelinated nerve fibers (139, 140) and CD11b+ myeloid cells (135). In favor of CTL-mediated cytotoxicity, in relapsing-remitting MS the granzyme levels in CSF were higher at relapse compared with the remission and healthy controls (138). To additionally corroborate the pathogenetic role of CTLs, neuroantigen-specific CD8+ T cells from MS patients and EAE mice were shown to be capable of killing neuronal cells and releasing IFN-γ and TNF-α *in vitro* (141–146). Consistently, a role of IFN-γ- and TNF-α co-producing CD8+ T (Tc1) cells in MS pathogenesis is assumed (146). Additionally, in active acute and chronic MS lesions high frequency of IL-17-producing CD8+ T (Tc17) cells (147, 148) was observed. These cells co-produce GM-CSF (149, 150), which contributes to myeloid cell activation and inflammation (151, 152). Besides, a positive correlation between the frequency of Tc17 cells in CSF and MS-related disability was found (153). This, in conjunction with data showing that co-transfer of Tc17 cells with subpathogenic numbers of CD4+ T cells could induce the disease in mice resistant to EAE and deficient in both IL-17-producing CD4+ T cells and Tc17 cells (154), corroborates the important role of Tc17 cells in EAE/MS pathogenesis. Data from a rat EAE model are in the same vein (155). In EAE mice Tc17 cells do not exhibit cytotoxicity (156) but have a high plasticity to convert into IFN-γ-producing cells with strong cytotoxic activity (157). In relapsing-remitting MS the frequency of IFN-γ/TNF-α co-producing Tc17 cells is higher in peripheral blood (PB) at relapse compared with the remission (158). Moreover, the rise in the count of PB effector/memory CD8+ T cells re-expressing CD45RA (TEMRA) and secreting high levels of cytotoxic molecules and proinflammatory cytokines (IFN-γ and TNF-α) speaks in favor of an active CD8+ T-cell response in MS (159).

Although majority of CD8+ T cells in active MS lesion are recruited from the periphery (160), CD8+ cells with features of tissue-resident memory T (Trm) cells are also present in these lesions (123, 161) and suggested to have an important role (162–166).

### Research Challenges

To investigate the contribution of CD8+ T cells in the development of NCI in MS considering the hypothesis that CD4+ T cells are involved in the initiation of brain lesions, whereas CD8+ T cells take on the leading role as the disease progresses.

### CD8+ T Cells in AD Lesions

Similar to MS, in AD (11, 12, 24, 25, 167) and several mouse models of AD (26, 27, 167, 168), CD8+ T cells were found to be the predominant type of T cells in the brain structures related to cognition. Additionally, a strong correlation between CD8+ T cell infiltration and tau pathology in both humans and experimental animals has recently been described (11, 27). They are located in close proximity to neuronal processes and microglia (12, 25, 167). More important, granules loaded with granzyme A were detected in CD8+ T cells from AD-affected hippocampi (12). To additionally corroborate the active role of CD8+ T cells in the disease pathogenesis, greater count of IFN-γ/TNF-α-producing CD8+ TEMRA cells was found in PB from patients diagnosed with mild neurocognitive disorder of AD type/AD patients (12). The latter correlated with NCI development (12). Of note, in PB from AD patients, higher frequency of Tc17 cells was detected than in controls (169). Moreover, a substantial proportion of CD8+ T cells in their intrathecal immune compartment belonged to a clonally expanded TEMRA subset (12).

Finally, CD8+ T cells with characteristics of Trm cells were also detected in hippocampi and subcortical white matter in AD patients and animal AD models (11, 26, 161, 165). However, their role in AD requires further research.

### Research Challenges

To elucidate the mechanistic immune signature of AD by exploring the role of CD8+ T cells in various experimental models of this disease.

## Putative Mechanisms of CD8+ T Cells Pathogenetic Action in the Development of Ms and Ad

Considering the aforementioned data, CD8+ T cells most likely contribute to the propagation of the initial lesions in AD and MS. It may be supposed that myeloid cells, primarily microglia, sense initial tissue damage (inflammatory CD4+ T cell-mediated and neurotoxic Aβ/phosphorylated tau protein-mediated damage in MS and AD, respectively), activate, upregulate MHC-I expression, and start the production of inflammasome-related cytokines and chemokines to recruit CD8+ T cells (124, 167, 170). In brain tissue CD8+ T cells may communicate with cells of distinct types, including neurons, which are shown to upregulate MHC-I expression in MS and AD (134, 171). Conceptually, CD8+ T cells may directly affect neuronal integrity acting via perforin-dependent delivery of several granzymes or via Fas-ligand/Fas receptor interactions (172, 173) and/or causing their “collateral” injury (174–176). They cause the “collateral” injury through destruction of myelin sheath and/or oligodendrocytes (174–176). Of note, CTLs are capable of sequential and simultaneous killing of several target cells, which is followed by “spillover” of cytotoxic molecules from immunological synapses and consequent collateral death of neighboring cells [reviewed in (177)]. Apart from neuron apoptosis, CTLs may cause their electrical silencing by increasing intracellular Ca2+ levels through massive insertion of channel-forming perforin (174, 178). The intracellular Ca2+ load may be augmented also by enhanced glutamate release from activated CD8+ T-cells and/or the target neurons themselves (174, 178–180). Apart from cell-to-cell contacts, CD8+ T cells could contribute to neuronal damage and NCI by releasing IFN-γ, TNF-α, and IL-17. These cytokines increase the permeability of blood-brain barrier and promote T-cell immigration into the CNS parenchyma (181, 182). Additionally, they upregulate MHC-I and thereby sensitize neurons/non-neural cells to CD8+ T cell (183). They could also trigger cell apoptosis (184). Moreover, in MS/EAE and AD/animal AD models these cytokines contribute to alterations in synaptic plasticity, and consequently NCI (185–187), by increasing glutamate release and/or affecting expression/phosphorylation of glutamate receptors (188–191).

Contrary to *in vitro* studies, a recent immunocytochemical study failed to show direct communication between CD8+ T cells and neurons/oligodendrocytes in MS, but pointed to their direct communication with distinct subsets of CD11b+ myeloid cells, including microglia, with manifold functional consequences (135). Specifically, CD8+ T cells may be activated to kill the target cells, whereas the activation of CD11b+ myeloid cells is associated with secretion of a broad array of proinflammatory mediators, including reactive oxygen and nitrogen species, and proinflammatory cytokines that damage neighboring cells, including neurons (135, 170, 192–194). The influence of CD8+ T cells on microglial secretion of proinflammatory cytokines and iNOS expression in chronic infection corroborates this notion (195). A recent study showed that in EAE mice CD8+ T cells communicate with brain infiltrating monocytes/monocyte-derived cells in a Fas-ligand/Fas receptor dependent manner (131). Such an interaction may also trigger the activation of microglia and consequently the expression of multiple genes encoding proinflammatory mediators (196). Thus, it may be speculated that CD8+ T-cell to myeloid cell communication in the brain represents a hotspot in the immunopathogenesis of MS. Besides, considering the localization of CD8+ T cells in AD-affected brain (12, 167), it may be assumed that they also contribute to AD progression by enhancing microglial activation. Specifically, the presumption is that with AD progression, as with MS/EAE progression (98), initially phagocyting microglia become more activated and consequently dysfunctional/damaging, i.e., that along the disease trajectory microglia function as a “double-edged sword” (12, 167).

Finally, given that Trm “sessile” cells were found among CD8+ T cells in AD, and particularly in relapsing-remitting and progressive MS (123, 161, 164–166, 197, 198) and that drugs inhibiting T-cell recruitment into the brain in MS showed limited therapeutic efficacy (123), the role of CD8+ Trm cells in the progression of MS and AD should be examined. Generally, they are characterized by upregulated expression of inhibitory receptors (PD-1 and CTLA-4) and diminished production of cytotoxic enzymes (123, 161, 164, 198). However, as they have preserved capacity to secrete proinflammatory cytokines (IFN-γ, TNF-α, and GM-CSF), they may contribute to myeloid cell activation when stimulatory signals (e.g., increased generation of proinflammatory mediators and toxic metabolites) overcome the inhibitory signals (123, 161, 164, 198). Hence, the increased brain levels of toxic soluble (o)Aβ variants in AD and possibly in MS may contribute to this activational/functional “switch” (199–202). Namely, the engagement of Toll-like receptor 2 on CD8+ Trm cells by toxic (o)Aβ variants may convert their partial activation to the full activation and proinflammatory cytokine production (199–202). Thus, it seems that the role of CD8+ Trm cells in MS (166) and possibly AD pathogenesis could be dependent on the disease stage. Altogether, it may be hypothesized that not only effector/memory CD8+ T cells recruited from the periphery but also CD8+ Trm cells contribute to the deleterious changes in the activational status/functional properties of microglia, and thereby to MS/AD perpetuation and NCI worsening.

### Research Challenges

To confirm the significance of CD8+ T cells and microglia communication in the progression of NCI in these diseases and particularly the contribution of CD8+ Trm cells to the detrimental output of this communication.

## Conclusions

**Figure 1:**
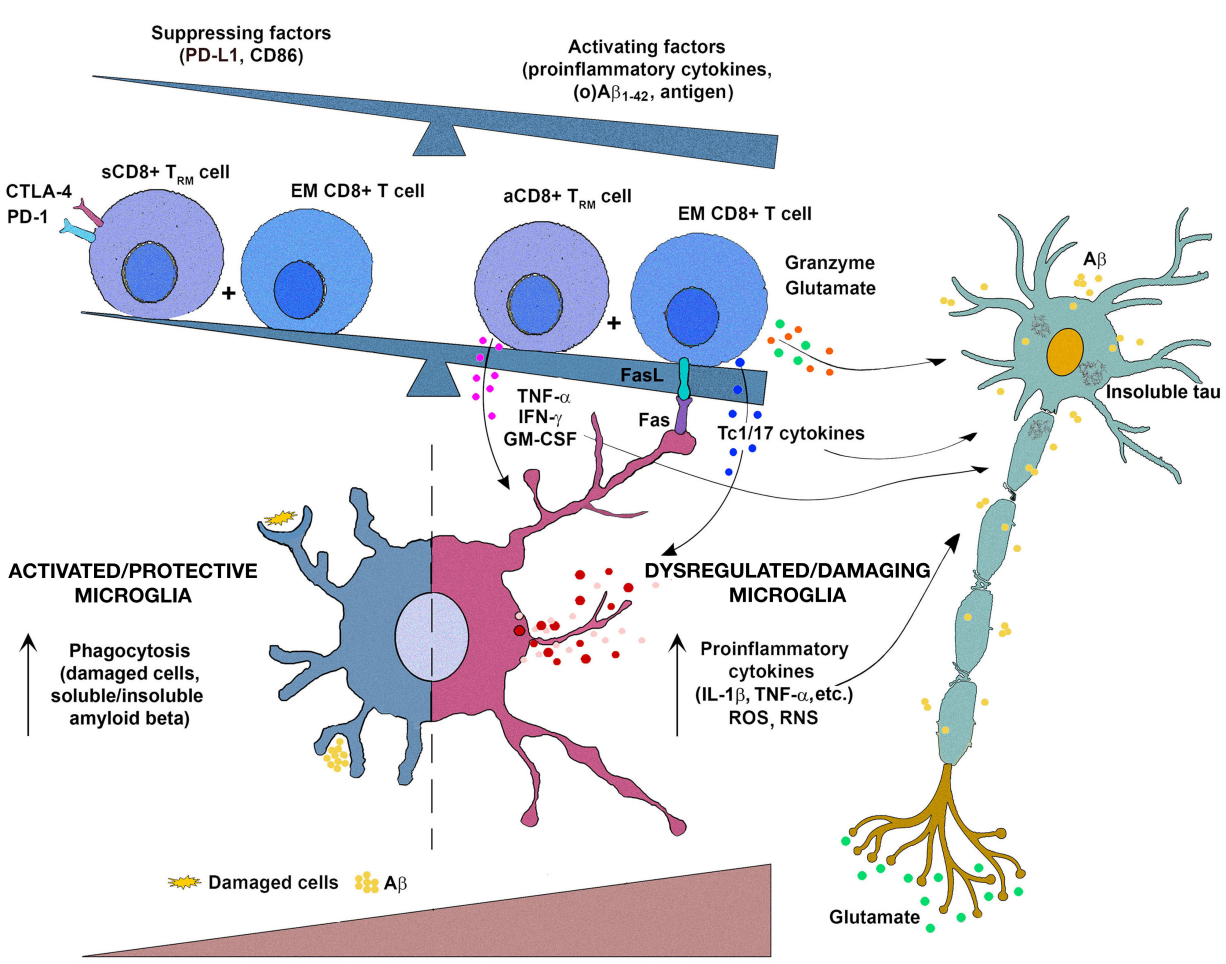
Schematic representation of the putative interactions between (re)activated CD8+ T cells and microglia in the progression of multiple sclerosis (MS) and Alzheimer's disease (AD). It may be hypothesized that upon entering in damaged (by CD4+ T cells and neurotoxic oligomeric amyloid β peptides [Aβ]/tau protein in MS and AD, respectively) brain tissue, effector/memory (EM) CD8+ T cells reactivate and additionally activate microglia through Fas ligand/Fas-mediated interactions and secretion of potentially damaging proinflammatory cytokines (IFN-γ, IL-17, TNF-α, GM-CSF). Consequently, microglia change their functional properties, viz. initially predominantly protective (phagocyting damaging cells, and Aβ variants and their soluble and insoluble assembly) microglia change to become dysfunctional/detrimental secreting damaging mediators, including proinflammatory cytokines (IL-1β, TNF-α), reactive oxygen species (ROS), and reactive nitrogen species (RNS) on the account of phagocyting ability. To this microglial transition also contribute CD8+ tissue-resident memory T (Trm) cells, as they in the response to alterations in the local microenvironment [mirrored in increasing accumulation of various activating mediators, e.g., proinflammatory cytokines, local metabolites, including Aβ_1−42_ and its soluble oligomers (o)Aβ_1−42_, which are shown to interact with TLR2 expressed on their cell surface] transit from a suppressed state (sCD8+ Trm cell) maintained by PD-L1- and CD86-mediated signaling to activated proinflammatory cytokine-secreting state (aCD8+ Trm cell). The damaging mediators derived from (re)activated EM CD8+ T cells (including glutamate, which is shown to contribute to activation of extrasynaptic NMDA receptors to promote cell death) and microglia, along with toxic metabolites/mediators from neurons themselves (Aβ, glutamate), contribute to neuron/neurite damage and further progression of the diseases.

It may be speculated that CD8+ T cells recruited from the periphery together with CD8+ Trm cells contribute to MS and AD progression acting on neurons/neurites not only directly but also indirectly by affecting the functional properties of microglia (Figure 1). This concept may serve as a basis for further research to formulate therapeutic interventions targeting not only effector/memory CD8+ T cells but also CD8+ Trm cells to moderate NCI severity. This could be particularly important, as patients with MS are now more likely than ever to enter old age and develop AD, so a number of individuals with these complex comorbidities is expected to increase (203).

## Author Contributions

ZS-V, SH, ON, and GL wrote the manuscript. All authors participated in data collection and interpretation, critically revised the manuscript, and approved the final version for submission. All authors contributed to the article and approved the submitted version.

## Conflict of Interest

The authors declare that the research was conducted in the absence of any commercial or financial relationships that could be construed as a potential conflict of interest.
